# The Predictive Value of Tumor Mutation Burden on Clinical Efficacy of Immune Checkpoint Inhibitors in Melanoma: A Systematic Review and Meta-Analysis

**DOI:** 10.3389/fphar.2022.748674

**Published:** 2022-03-09

**Authors:** Biao Ning, Yixin Liu, Miao Wang, Yi Li, Tianzi Xu, Yongchang Wei

**Affiliations:** ^1^ Department of Radiation and Medical Oncology, Zhongnan Hospital of Wuhan University, Wuhan, China; ^2^ Hubei Key Laboratory of Tumor Biological Behaviors Zhongnan Hospital of Wuhan University, Wuhan, China; ^3^ Hubei Cancer Clinical Study Center Zhongnan Hospital of Wuhan University, Wuhan, China

**Keywords:** tumor mutation burden, immune checkpoint inhibitor, OS, PFS, melanoma, meta-analysis

## Abstract

**Background:** Tumor mutational burden (TMB) is a genomic biomarker that can predict favorable responses to immune checkpoint inhibitors (ICIs). Although we have better understanding of TMB in cancer immunity and cancer immunotherapy, the relationship between TMB and the clinical efficacy of ICIs remains unknown in the treatment of melanoma patients. Here, we conduct a systematic review and meta-analysis to evaluate the predictive value of TMB on the efficacy of ICIs in patients with melanoma.

**Methods:** We systematically collected data from PubMed, Embase, Cochrane Library, CNKI, China Biomedical Database (CBM), and Wanfang Database. The end date was set to 26 June 2021. We included retrospective studies or clinical trials of ICIs that reported hazard ratios (HRs) for overall survival and/or progression-free survival according to TMB. Data for 1,493 patients from 15 studies were included. In addition, pooled effect size, heterogeneity analysis, sensitivity analysis, publication bias detection, and subgroup analysis were performed based on the included data.

**Results:** Patients with high TMB showed significantly improved OS (HR = 0.49, 95% CI: 0.33, 0.73; *p* = 0.001) and PFS (HR = 0.47, 95% CI: 0.33, 0.68; *p* < 0.001) compared with patients with low TMB. This association was very good in patients treated with monotherapy, that is, anti-CTLA-4 or anti-PD-(L)-1 inhibitors, but not for the patients treated with a combination of the two drugs. The subgroup analysis results showed that heterogeneity was substantial in the targeted next-generation sequencing (NGS) group. Publication bias was detected, and the results were visualized using the funnel chart. And sensitivity analysis and trim-and-fill method analysis showed that our results were stable and reliable.

**Conclusion:** High TMB is associated with improved OS and PFS in melanoma patients treated with mono-drug ICIs. TMB determined by NGS should be standardized to eliminate heterogeneity. Therefore, the role of TMB in identifying melanoma patients who may benefit from ICI should be further determined in more randomized controlled trials in the future.

## Introduction

As a highly aggressive type of skin cancer, melanoma is the leading cause of skin cancer-related deaths, causing nearly 60,000 deaths worldwide each year (Karimkhani et al., 2017). The results of the latest global cancer statistics in 2020 showed that there were more than 320,000 new skin melanoma patients and more than 57,000 deaths (Sung et al., 2021). At present, the basic principle of clinical treatment of melanoma is extensive local surgical resection, but the outstanding feature of melanoma is that it is prone to distant metastasis in the early stage of onset. Meantime, sensitivity to traditional radiotherapy and chemotherapy of melanoma is very low, and drug resistance is prone to occur. Due to the poor efficacy of existing programs, the 5-year survival rate of melanoma patients is less than 10% (Kaufman et al., 2018; Franke and van Akkooi, 2019).

With the deepening of research, the emergence of immune checkpoint inhibitors (ICIs) has completely changed the treatment prospects for patients with stage III/IV melanoma. A variety of ICIs have been proven to have a good effect on patients with unresectable or metastatic melanoma. These drugs, whether used as a monotherapy or in combination, produce lasting improvement in survival rates and potential cure rates for patients with advanced-stage III and IV melanomas (Eggermont et al., 2016; Schachter et al., 2017; Larkin et al., 2019). However, it should be noted that not all melanoma patients can benefit from immunotherapy (Lugowska et al., 2018). Melanoma patients urgently need effective biomarkers that can indicate the potential benefits of immunotherapy.

Tumor mutational burden (TMB) is defined as the total number of somatic mutations per megabase or the non-synonymous mutations in tumor tissues, including replacement and insertion–deletion mutations, and it is likely to be a promising biomarker. According to reports, in melanoma, non-small-cell lung cancer and urothelial cell carcinoma patients with high TMB had a better response to ICI and survival rate than patients with low TMB (Rosenberg et al., 2016; Teo et al., 2018; Alborelli et al., 2020; Huang et al., 2020; Valero et al., 2021a; Gogas et al., 2021). Meanwhile, Liu et al. pointed out that compared with copy number alteration (CNA) alone, CNA combined with TMB as a new biomarker showed better prediction for ICI efficacy (L. Liu D. et al., 2019). Furthermore, TMB is also a potential predictor for ICI efficacy in liver cancer and biliary tract cancer (Rizzo and Brandi, 2021; Rizzo and Ricci, 2021; Rizzo et al., 2021). However, TMB, as a biomarker, still has some controversy in the evaluation of the outcome of melanoma patients with ICI therapy. Therefore, we conducted a comprehensive systematic review and meta-analysis to evaluate the effect of TMB on the efficacy of immune checkpoint inhibitors in melanoma patients, and overall subgroup analysis and sensitivity analysis to identify potential sources of heterogeneity.

## Materials and Methods

### Literature Search

This meta-analysis follows the PRISMA statement (Moher et al., 2009). As of 26 June 2021, systematic literature searches had been conducted on PubMed, Embase, Cochrane Library, China Knowledge Network (CNKI), China Biomedical Database (CBM), and Wanfang Database. The search terms were (“Immune Checkpoint Inhibitors” OR “Immune Checkpoint Inhibitor” OR “Immune Checkpoint Blockers” OR “Immune Checkpoint Blockade” OR “Immune Checkpoint Inhibition” OR “PD-L1 Inhibitors” OR “PD-L1 Inhibitor” OR ″ Programmed Death-Ligand 1 Inhibitors” OR “CTLA-4 Inhibitors” OR “CTLA-4 Inhibitor” OR “Cytotoxic T-Lymphocyte-Associated Protein 4 Inhibitors” OR “Cytotoxic T-Lymphocyte-Associated Protein 4 Inhibitor” OR “PD-1 Inhibitors” OR “PD-1 Inhibitor” OR “Programmed Cell Death Protein 1 Inhibitor” OR “Programmed Cell Death Protein 1 Inhibitors” OR “PD-1-PD-L1 Blockade” OR “Ipilimumab” OR “Tremelimumab” OR “Nivolumab” OR “Pembrolizumab” OR “Lambrolizumab” OR “Atezolizumab” OR “Avelumab” OR “Durvalumab”) AND (“Melanoma” OR “Melanomas” OR “Malignant Melanoma” OR “Malignant Melanomas”) AND (“mutation burden” OR “mutational Burden” OR “mutation load” OR “mutational load” OR “TMB” OR “TML”) and the corresponding Chinese search terms. In addition, we manually searched the references of selected articles to obtain all possible relevant studies. All searched documents were not restricted to languages.

### Literature Inclusion and Exclusion Criteria

In order to meet the conditions, the study must meet the following inclusion criteria: 1) pathologically confirmed melanoma; 2) cohort studies or clinical trials used TMB with cutoff values to evaluate the clinical outcome of melanoma patients who were treated with PD-1/PD-L1, CTLA-4, or their combined inhibitor; 3) hazard ratio (HR) of overall survival (OS) or progression-free survival (PFS), and their 95% confidence interval (95% CI) were given in the article, or there were enough data to calculate them; and 4) the number of evaluable patients was not less than 20. Exclusion criteria were as follows: insufficient information and data, non-original research (such as reviews and meta-analysis), repeated research, letters, editorials, comments, conference abstracts, and case reports.

### Data Extraction

Two researchers independently extracted data from the included studies, and any inconsistencies were resolved through consultation with all researchers. The following information were extracted from each study: title, first author, year of publication, study type, sample size evaluable for TMB, immune checkpoint inhibitor category, TMB sequencing method, TMB cutoff value, and results (PFS, OS).

### Literature Quality Evaluation

The Newcastle–Ottawa Scale (NOS) quality assessment scale was used to assess the quality of the included studies or cohorts (Stang, 2010). The total score ranged from 0 to 9, with 8-9 points indicating high research quality, 5-7 points indicating medium quality, and less than 5 points indicating poor research quality.

### Statistical Methods and Data Analysis

The main goal of this meta-analysis was to compare the efficacy of ICIs between the TMB high group and the TMB low group, measured by HR of OS or PFS. If the chi-square test *p* < 0.1 or I^2^>50%, it was considered that there was significant heterogeneity among included studies (Higgins et al., 2003). If heterogeneity was significant, the random-effects model was used to reduce the impact of heterogeneity on the results, otherwise, the fixed-effects model was used. In addition, a funnel chart was constructed, and Begg’s test and Egger’s test were performed to assess publication bias (*p* > 0.1 was considered to have no obvious publication bias). On the other hand, the trim-and-fill method analysis and sensitivity analysis were used to test the result’s stability of our meta-analysis. In order to further explore the source of heterogeneity, a subgroup analysis was performed based on immune checkpoint inhibitor type, TMB sequencing method, study type, and the number of patients included in the study. STATA15.1 software was used for statistical analysis.

In addition, several articles provided raw data or graphs but did not report HR values and 95% CI. For original survival data, SPSS23.0 was used to calculate the HR value and 95% CI using the Cox proportional hazard regression model. For the Kaplan–Meier curve, Engauge Digitizer was used to extract survival data from the graph, and then HR value was estimated using the method reported by Tierney et al. (2007).

## Results

### Literature Searching and Results Screening

**FIGURE 1:**
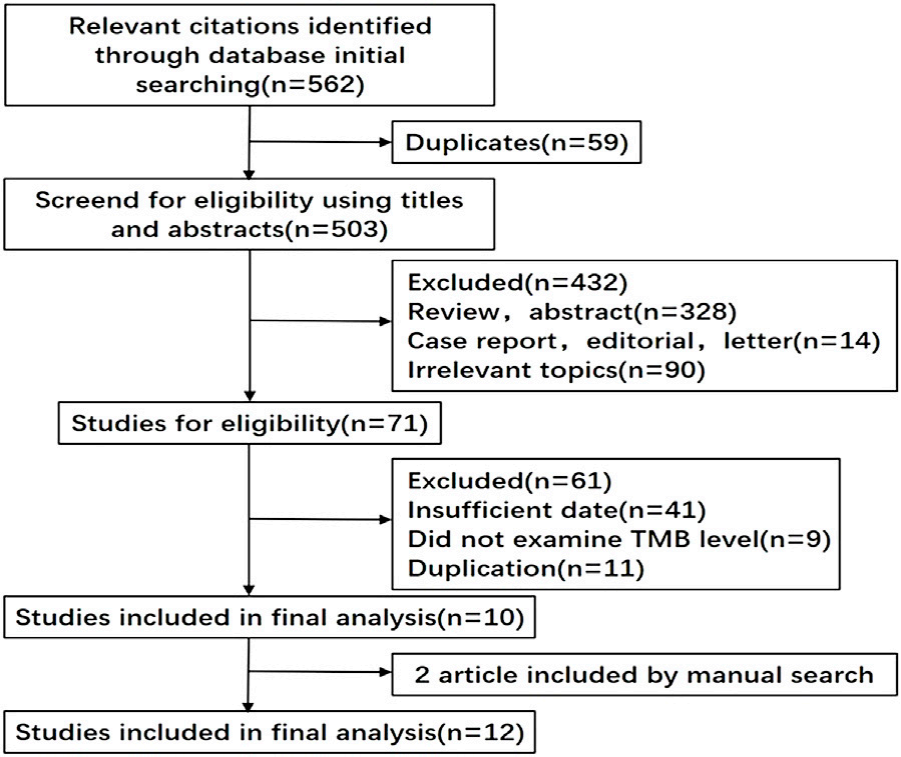
Flowchart of literature search and screening.

We initially retrieved 562 records with the keywords. Excluding 59 duplicate documents, we screened the remaining 503 documents by reading the titles and abstracts, and then only 71 documents were left. After reading the full-text 12 documents including fifteen studies were eligible for the final analysis. The publication year ranged from 2014 to 2021, and the number of patients in each study ranged from 23 to 298, with a total of 1,493 patients (Figure 1) (Snyder et al., 2014; Van Allen et al., 2015; Johnson et al., 2016; Roszik et al., 2016; Hugo et al., 2016; Cristescu et al., 2018; Morrison et al., 2018; Hamid et al., 2019; Liu L. et al., 2019; Yusko et al., 2019; Gogas et al., 2021; Valero et al., 2021b).

### Basic Situation and Quality Assessment of Selected Documents

The basic characteristics of the studies included in our study are shown in Table 1 and Appendix Supplementary Table S1 in the Supplementary Material. And results of the NOS quality assessment scale are shown in Table 2. Eight studies had high quality, and the rest showed medium quality, which ensured a high quality of the included studies and improved the reliability of our meta-analysis.

**TABLE 1:** Basic characteristics of included studies.

Study	Experimental drugs	TMB cutoff value	Detection method	Type of study	Number of participants	Outcomes
Van Allen (2015)	Anti-CTLA-4	197	WES	Retrospective cohort	110	PFS, OS
Snyder (2014a)	Anti-CTLA-4	100	WES	Retrospective cohort	25	OS
Snyder (2014b)	Anti-CTLA-4	100	WES	Retrospective cohort	39	OS
Roszik (2016)	Anti-CTLA-4	100	Targeted NGS	Retrospective cohort	76	OS
Morrison (2018)	Anti-PD-1/anti-CTLA-4	7.1 muts/Mb	Targeted NGS	Retrospective cohort	160	OS
Cristescu (2018)	Anti-PD-1	191.5	WES	Clinical trial	89	PFS
Johnson (2016)	Anti-PD-1/PD-L1	High: 23.1 muts/Mb; low:3.3 muts/Mb	Targeted NGS	Retrospective cohort	65	PFS, OS
Hugo (2016)	Anti-PD-1	489	WES	Retrospective cohort	38	OS
Liu (2019)	Anti-PD-1	6.5 muts/Mb	WES	Retrospective cohort	144	PFS, OS
Hamid (2019)	Anti-PD-L1	16/MB	Targeted NGS	Clinical trial	23	PFS, OS
Yusko (2019a)	Anti-PD-1/anti-CTLA-4	171	WES	Clinical trial	30	OS
Yusko (2019b)	Anti-PD-1/anti-CTLA-4	159	WES	Clinical trial	38	OS
Gogas (2021a)	Anti-PD-L1	10 muts/Mb	Targeted NGS	Clinical trial	179	PFS
Gogas (2021b)	Anti-PD-1	10 muts/Mb	Targeted NGS	Clinical trial	179	PFS
Valero (2021)	ICI	Top 20th percentile	Targeted NGS	Retrospective cohort	298	OS

TMB: tumor mutation burden; OS: overall survival; PFS: progression-free survival; WES: whole exome sequencing; NGS: next-generation sequencing; muts: mutations.

**TABLE 2:** NOS scores of 15 studies.

Study	Q1	Q2	Q3	Q4	Q5	Q6	Q7	Q8	Total NOS score
Van Allen (2015)	1	1	1	1	1	1	1	1	8
Snyder (2014a)		1	1	1		1	1	1	6
Snyder (2014b)		1	1	1		1	1	1	6
Roszik (2016)	1	1	1	1	1	1	1	1	8
Morrison (2018)	1	1	1	1		1	1	1	7
Cristescu (2018)	1	1	1	1	2	1	1	1	9
Johnson (2016)	1	1	1	1		1	1	1	7
Hugo (2016)		1	1	1	1	1	1	1	7
Liu (2019)	1	1	1	1		1	1	1	7
Hamid (2019)	1	1	1	1	2	1	1	1	9
Yusko (2019a)	1	1	1	1	2	1	1	1	9
Yusko (2019b)	1	1	1	1	2	1	1	1	9
Gogas (2021a)	1	1	1	1	2	1	1	1	9
Gogas (2021b)	1	1	1	1	2	1	1	1	9
Valero (2021)	1	1	1	1		1	1	1	7

Q1: representativeness of the exposed cohort; Q2: selection of the non-exposed cohort; Q3: ascertainment of exposure; Q4: outcome of interest not present at the start of the study; Q5: comparability of cohorts; Q6: assessment of outcome; Q7: follow-up long enough; Q8: adequacy of follow up of cohorts. And the NOS assigns up to a maximum of nine points for the least risk of bias in three domains.

### Analysis of the Relationship Between TMB and ICI Efficacy

**FIGURE 2:**
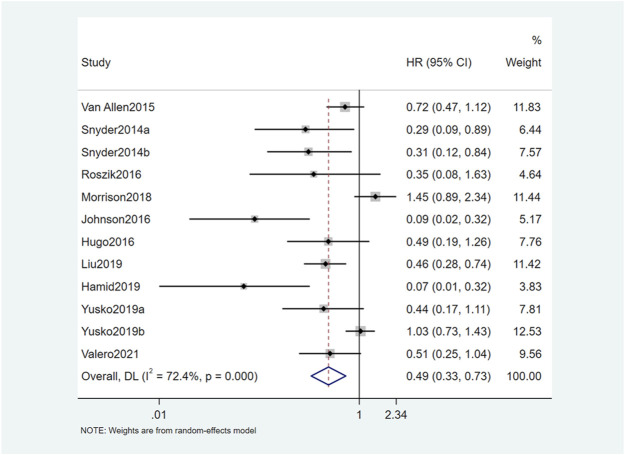
Forest plot of association between TMB and OS.

**FIGURE 3:**
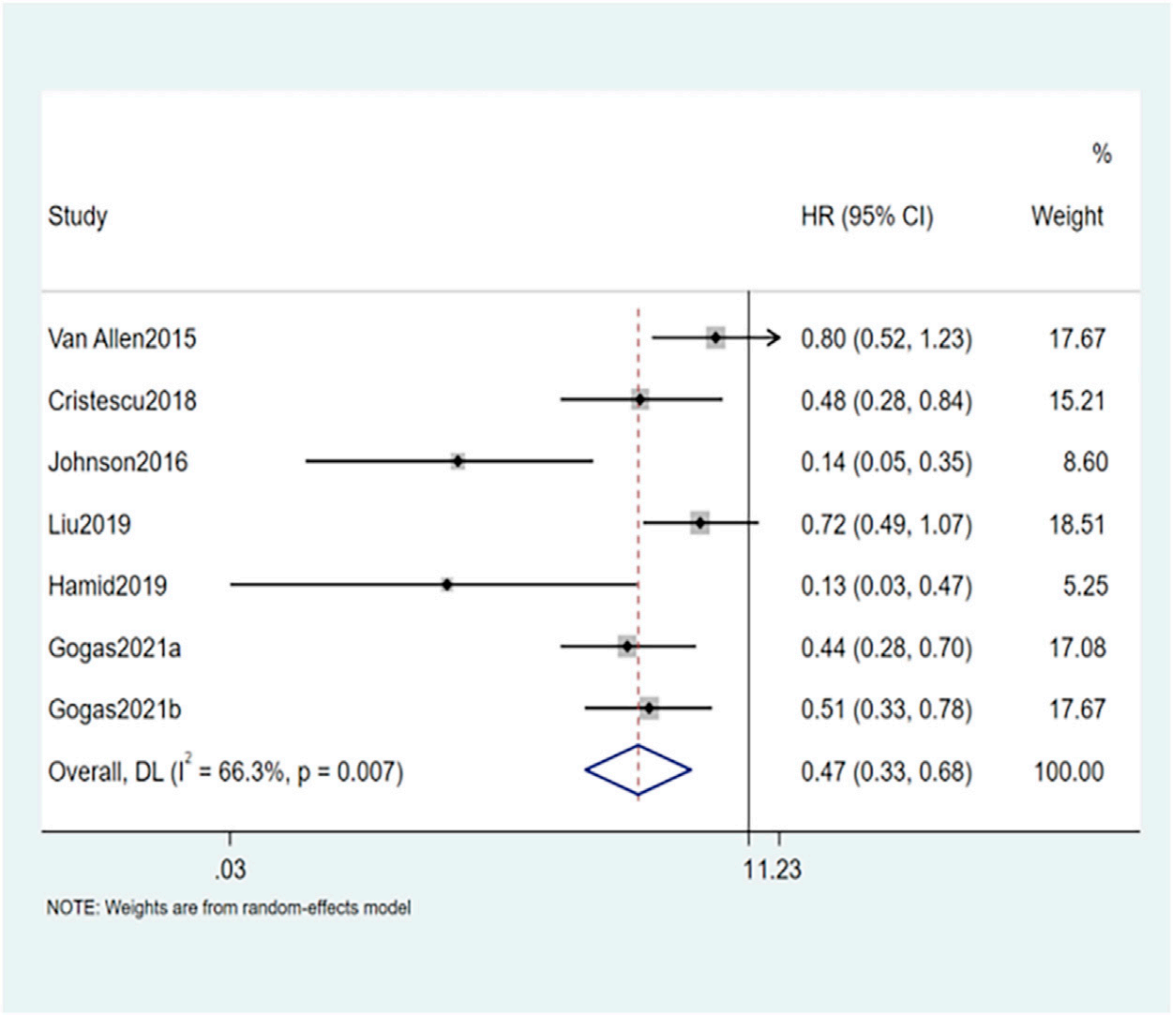
Forest plot of association between TMB and PFS.

The results of this study showed that OS and PFS were significantly improved in patients with high TMB. A total of 12 studies reported a relationship between TMB and OS. The OS of patients with high TMB was significantly better than patients with low TMB (HR = 0.49, 95% CI: 0.33, 0.73; *p* = 0.001) (Figure 2). Seven studies reported the relationship between TMB and PFS, and the result was similar to OS. The PFS of TMB high group was significantly improved (HR = 0.47, 95% CI: 0.33, 0.68; *p* < 0.001) (Figure 3). Obvious heterogeneity could be observed in the two groups, OS group (I^2^ = 72.4%, *p* = 0.001) and PFS group (I^2^ = 66.3%, *p* < 0.001). Therefore, both groups used the random-effects model to pool effect sizes to reduce the impact of heterogeneity on results.

### Publication Bias

**FIGURE 4:**
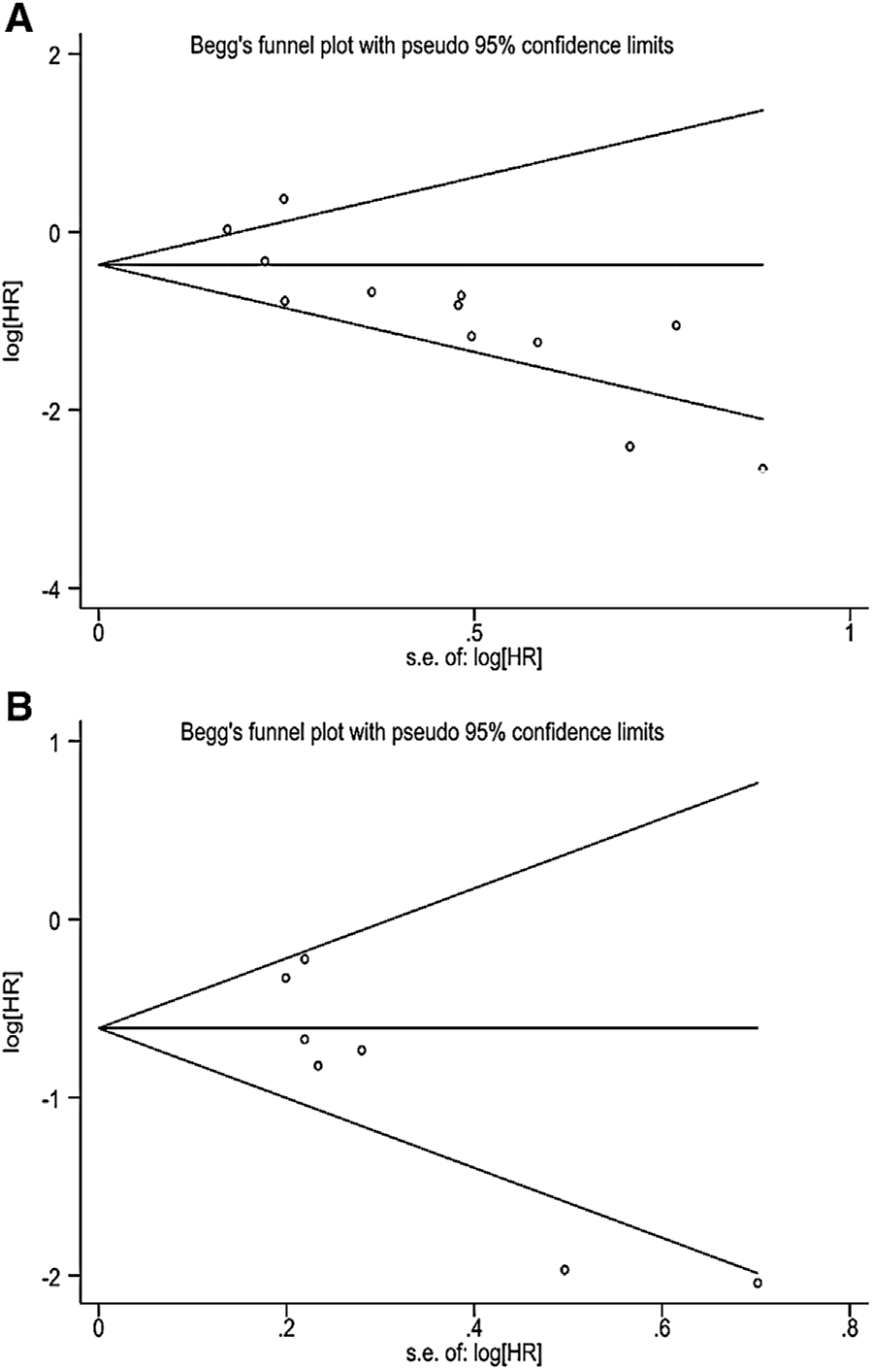
**(A)** Begg’s funnel plot of correlation between TMB and OS. **(B)** Begg’s funnel plot of correlation between TMB and PFS.

**FIGURE 5:**
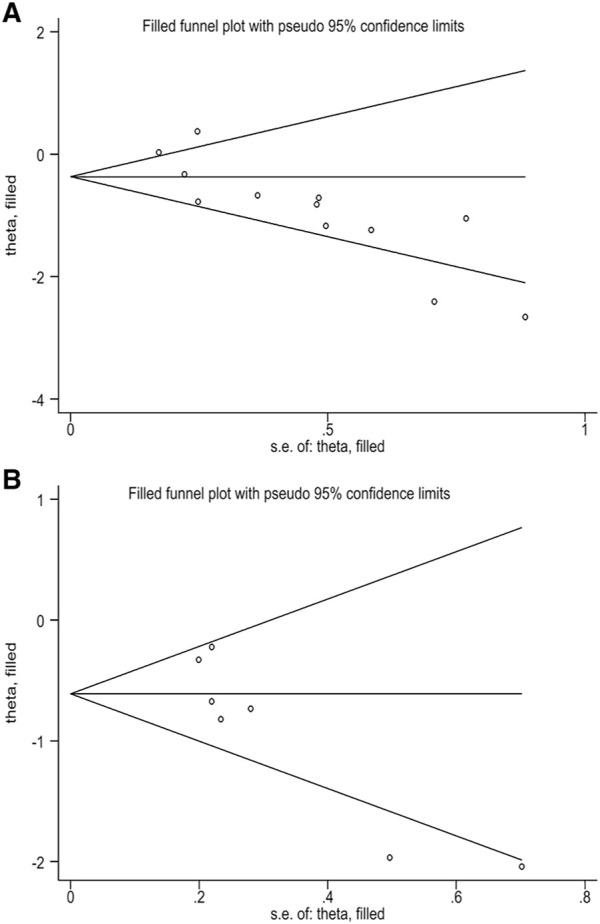
**(A)** Filled funnel plot of correlation between TMB and OS. **(B)** Filled funnel plot of correlation between TMB and PFS.

This study evaluated publication bias by analyzing Egger’s test and Begg’s test. There was evidence of publication bias, with asymmetry in the funnel plots (Figures 4A,B). And Egger’s test *p*-values were, respectively, 0.002 and 0.012. The trim-and-fill method analysis resulted that no studies were clipped or new studies were added, suggesting that the result of our study was stable and reliable (Figures 5A,B).

### Sensitivity Analysis and Subgroup Analysis

Sensitivity analysis showed a good stability of the pooled HR. Relatively speaking, Morrison,^[22]^ Johnson,^[24]^ and Hamid ^[27]^ had high heterogeneity. After excluding the three documents, I^2^ of the OS group dropped to 48.7%, HR = 0.56 (95% CI: 0.40, 0.77; *p* < 0.001); And I^2^ of the PFS group dropped to 27.0%, HR = 0.59 (95% CI: 0.46, 0.74; *p* < 0.001) (Appendix Supplementary Figure S1, 2 in the Supplementary Material).

The results of subgroup analysis are shown in Table 3. For different treatment strategies, when anti-CTLA-4 or anti-PD-(L)1 was used alone, the OS and PFS of patients with high TMB were dramatically better than patients with low TMB. However, when combining these two drugs, the OS and PFS showed no difference between the two groups. Second, a subgroup analysis of TMB detection methods showed that regardless of whether WES or targeted NGS, patients with high TMB were associated with prolonged OS and PFS. Compared with the WES detection method, the targeted NGS method showed obvious heterogeneity. In addition, subgroup analysis was performed on the study type and number of participants in each study. The correlation between the TMB level and OS was not statistically different among the clinical trial group and the number of participants ≥100 group, while the correlation between the TMB level and PFS was not statistically different in the cohort group (Appendix Supplementary Figure S3–10 in the Supplementary Material).

**TABLE 3:** Subgroup analysis of predictive value of TMB for ICI treatment on melanoma.

Category Experimental drugs	HR (95%CI)	OS I^2^ (%)	P	HR (95%CI)	PFS I^2^ (%)	P
Anti-CTLA-4	0.48 (0.28.0.81	31.0	0.006		NA	
Anti-PD-(L)1	0.25 (0.10.0.60)	65.3	0.002	0.42 (0.29.0.63)	64.1	<0.001
Others*	0.85 (0.53.1.38)	65.2	0.519		NA	
**Detection method**						
WES	0.56 (0.39.0.82)	58.1	0.003	0.68 (0.52.0.89)	7.9	0.005
Targeted NGS	0.33 (0.11.0.99)	84.3	0.048	0.32 (0.18.0.57)	64.4	<0.001
**Type of study**						
Cohorts	0.49 (0.31.0.77)	69.2	0.002	0.50 (0.25.1.03)	81.3	0.061
Clinical trials	0.41 (0.12.1.41)	82.0	0.156	0.45 (0.33.0.60)	14.6	<0.001
**Number of participants**						
<100 participants	0.33 (0.17.0.66)	74.2	0.001	0.23 (0.09.0.61)	69.3	0.003
≥100 participants	0.72 (0.43.0.20)	75.4	0.208	0.61 (0.46.0.79)	38.0	<0.001

Non-monotherapy: NA: not available.

## Discussion

The primary target of this meta-analysis was to assess the association between TMB and OS or PFS in melanoma patients treated with ICIs. Our pooled analysis integrated the data of 1,493 melanoma patients, and results showed that compared with the low-TMB group, the risk of death in the high-TMB group was reduced by 51%, and the risk of disease progression was reduced by 53%. In patients receiving treatment other than ICIs, no such difference in survival based on TMB levels was found (Cao et al., 2019). Since most of the studies we included were conducted in Western countries, the role of TMB in Asian melanoma patients needs more relevant clinical studies. In addition, the results of this article showed that TMB was clinically significant in melanoma patients who were treated with a single ICI, while the result in the non-monotherapy group was opposite. Due to an insufficient number of patients included in our study, whether TMB could predict the efficacy of combination therapy (anti-PD-(L)1 plus anti-CTLA-4) in melanoma patients required more studies to further confirm.

TMB, a quantitative biomarker, is defined by the total number of somatic mutations in the coding region of genes in tumor cells, which may reflect both mutation status and neoantigen load (Zehir et al., 2017). Neoantigen load has been shown to be associated with the clinical response to immunotherapy in several studies (Rizvi et al., 2015; Rooney et al., 2015). Although TMB is not completely equivalent to the neoantigen load produced via many mechanisms, tumors harboring more mutations generate more neoantigens and have a greater likelihood of being recognized by the immune system. When the PD-1/PD-L1 pathway is activated, it can inhibit the proliferation of T lymphocytes and suppress the immune function of T cells (Sharpe and Pauken, 2018). Thus, TMB can reasonably be assumed to be a proxy for neoantigen load to predict the response to immunity therapy.

The quantitative detection of TMB currently uses mainly WES and the targeted NGS method. Interestingly, in our subgroup analysis, whether for OS or PFS, significant heterogeneity was concentrated in the targeted NGS group; and sensitivity analysis found that the three documents with greater heterogeneity all detected TMB by the using targeted NGS method. So different TMB sequencing methods might clarify most of the heterogeneities. Currently, simultaneous detection of tumors and matched blood or normal tissue using WES is considered the gold standard for TMB quantification, and many initial studies have been performed on this method (Snyder et al., 2014; Hugo et al., 2016; Riaz et al., 2017). However, measuring TMB by WES has some limitations in daily clinical practice due to the tissue processing difficulty, time- and labor-intensiveness due to its large sequencing capacity, and subsequent high costs. With relatively cheap costs and simple operation, NGS panels consisting of only hundreds of genes are more suitable for clinical needs than WES (Horak et al., 2016). So far, the FDA has approved two targeted NGS panels for TMB analysis: Memorial Sloan Kettering-Integrated Mutation Profiling of Actionable Cancer Targets (MSK-IMPACT) panel and Foundation One CDx (F1CDx) panel. However, our results showed that there was significant heterogeneity in different targeted NGS panels, which might affect the accuracy and stability of TMB prediction. In fact, panel-based TMB evaluation is also affected by several experimental factors (e.g., tumor purity or sequencing depth) and variant calling pipeline, which need to be standardized among different targeted NGS panels (Chan et al., 2019; Fancello et al., 2019).

One of the most critical issues of TMB is the optimal threshold for predicting the effect of immunotherapy. As TMB varies greatly in different tumors, there may not be a universal TMB cutoff value for all cancer types, especially cancers with high TMB levels, such as NSCLC and melanoma (Chalmers et al., 2017; Samstein et al., 2019). In order to determine the best TMB cutoff value, a Foundation Medicine officially divided TMB into three groups: low (1–five Mut/Mb), intermediate (6–19 Mut/Mb), and high (≥20 Mut/Mb) (Goodman et al., 2017). In addition, in 2020, based on the results of the Keynote158 trial, the FDA approved anti-PD-1 therapy for any type of solid tumor with TMB ≥10 mut/Mb (Marabelle et al., 2020). Although studies have confirmed that patients with TMB ≥10 mut/Mb have generally higher response rates to ICI treatment in many tumors (Valero et al., 2021a), a new study suggested that simply defining a certain threshold value as “high TMB” was not suitable for predicting the effect of immunotherapy for each type of tumor (McGrail et al., 2021). In our meta-analysis, the detection of TMB in each study was based on different detection technologies and different gene panel platforms, in addition, TMB may come from tissue or blood samples, these factors would all affect the optimal TMB cutoff value. Nevertheless, while TMB testing has important guiding significance in immunotherapy strategies, a number of clinical trials are in progress in the context of TMB assessment in diverse cancers (Chan et al., 2019), and these trials are expected to provide more high-quality data to help us determine appropriate TMB cutoff value for certain cancer types. In addition, according to a recent study, Vega et al. developed a calibration tool based on panel assays from 16 participating laboratories which will help improve the consistency and reliability of panel tissue TMB estimation across platforms and facilitate the use of this complex biomarker in clinical decision making (Vega et al., 2021).

Although the result of our study suggested that TMB was indeed related to the efficacy of immunotherapy and had good predictive value in some patients, not all patients with high TMB could benefit from it. The expression of programmed cell death receptor 1 ligand (PD-L1) is another major biomarker of response to ICIs. However, most randomized clinical trials have confirmed that PD-L1 expression remains only moderately predictive, being dynamic, heterogeneous, and unable to distinguish adaptive and constitutive patterns of expression and neglecting variable characteristics of the tumor immune microenvironment (Chan et al., 2019). According to previous reports, other biomarkers, such as lactate dehydrogenase (LDH) and driver mutations in NRAS and NF1, although related to the response to ICIs, could not independently predict benefit to ICIs (Johnson et al., 2015; Axelrod et al., 2018; Wagner et al., 2018). In this context, we propose to combine these different biomarkers for evaluation to develop a multivariate prediction model and scoring system (Hur et al., 2016; Zhai et al., 2017; Beukinga et al., 2018; Jiang et al., 2020). Moreover, Langen et al. summarized the potential value of positron emission tomography (PET) in predicting and evaluating the treatment response to immunotherapy (Dimitrakopoulou-Strauss, 2019; Borm et al., 2021). Therefore, other biomarkers like FDG PET-CT or PD-1 or PD L-1 imaging in combination with TMB and laboratory parameters should be used in the future (as prognostic markers and also for therapy monitoring) to get a holistic approach of tumor biology including heterogeneity of the metastatic disease and in order to tailor therapy on a personalized basis.

In addition, this meta-analysis has some limitations. First of all, there were differences in the sample size of included studies, leading to large differences in the sample size between different subgroups. Some studies with small sample sizes may be the main source of publication bias in this meta-analysis. Second, the HRs and corresponding 95% CIs were not reported in some studies, and we were unable to obtain their original data; therefore, these studies were excluded, which may also lead to potential publication bias. Third, some important clinical features, which were reported to be related to the efficacy of ICIs, such as age and gender (Chalmers et al., 2017; Conforti et al., 2018; Wu et al., 2019), have been ignored due to insufficient data. Finally, TMB is still a controversial biomarker in clinical practice. There are still many problems in the standardization of TMB detection. In the future, more relevant studies are needed to define and clarify the optimal TMB threshold.

Overall, this study is the first large-sample meta-analysis on the effect of TMB in the efficacy of ICI therapy in patients with melanoma, which is of important reference value for future research studies on the relationship between TMB and immunotherapy and the clinical application of TMB in melanoma patients. Although there are limitations, we conducted subgroup analysis from some aspects and found most of the sources of heterogeneity. In addition, sensitivity analysis and trim-and-fill method analysis showed that our results have good stability. Therefore, despite the current technical and practical obstacles, we believe that after the standardized TMB cutoff value is determined in the future, it may become a preferable biomarker for screening melanoma patients who are most suitable for ICI treatment. Additionally, we consider that a comprehensive prediction model of multiple biomarkers (such as TMB, PD-L1, LDH, and PET) may be beneficial. Finally, we also take the view that TMB may be used as a predictor not only in ICI therapy but also play a predictive role in new immunotherapies, such as therapeutic vaccines and chimeric antigen receptor T-cell therapy.

## Conclusion

Our meta-analysis result shows that high TMB can predict the improvement of ICI efficacy in patients with melanoma, indicating that TMB can be used as a new potential predictive biomarker for mono-immunotherapy strategy in melanoma. In the future, more large-sample, standardized design studies are needed to further verify the predictive value of TMB in subgroups, such as combination therapy (anti-PD-(L)1 plus anti-CTLA-4). In addition, the clinically targeted NGS used to quantify TMB should be standardized to eliminate the influence of heterogeneity. Finally, combining TMB with eligible biomarkers may expand the choice of patients who will benefit from immune checkpoint inhibitors.

## Data Availability

The original contributions presented in the study are included in the article/Supplementary Material, further inquiries can be directed to the corresponding author.
