# Supplementary material for: The Predictive Value of Tumor Mutation Burden on Clinical Efficacy of Immune Checkpoint Inhibitors in Melanoma: A Systematic Review and Meta-Analysis
Source: Front Pharmacol. 2022 Mar 9;13:748674. doi: 10.3389/fphar.2022.748674 (PMC8959431; doi:10.3389/fphar.2022.748674)
Supplement: Supplementary file 2 [file Table2.DOCX]

Supplementary Table 1. Patient demographics and baseline characteristics

| **Clinical characteristics** | **Gogas2021** | **Van Allen2015** | **Snyder2014** | **Roszik2016** | **Morrison2018** | **Cristescu2018** | **Johnson2016** | **Hugo2016** | **Liu2019** | **Hamid2019** |
| --- | --- | --- | --- | --- | --- | --- | --- | --- | --- | --- |
| **Median age (range), y** | 66 (54-73) | 61.5(18-86) | 61.5 (18-90) | 59.5(18-77) | NA | 60 (30-88) | 58.5 (32-85) | 61.5 (19-84) | NA | 63 (21-83) |
| **Male, n (%)** | 270 (60.5) | 78 (70.9) | 39 (60.9) | 57 (75.0) | 107 (66.9) | 58 (65.2) | 38 (58.5) | 27 (71.1) | 84 (58.3) | 30 (66.7) |
| **Stage at screening, n (%) of patients with cutaneous histology** |  |  |  |  |  |  |  |  |  |  |
| M1a | NA | 6 (5.5) | 6 (9.4) | 19 (25.0) | NA | NA | 5 (7.7) | 5 (13.2) | 8 (5.6) | 5 (14.0) |
| M1b | NA | 16 (14.5) | 11 (17.2) | 18 (23.7) | NA | NA | 9 (13.8) | 4 (10.5) | 18 (12.5) | 5 (14.0) |
| M1c | 174 (39.0) | 78 (70.9) | 44 (68.8) | 36 (47.4) | NA | NA | 51 (78.5) | 28 (73.7) | 108 (75.0) | 25 (71.0) |
| **LDH elevated** | 111 (24.9) | 48 (43.6) | 13 (20.3) | 14 (18.4) | NA | NA | NA | NA | 71 (49.3) | 7 (15.6) |


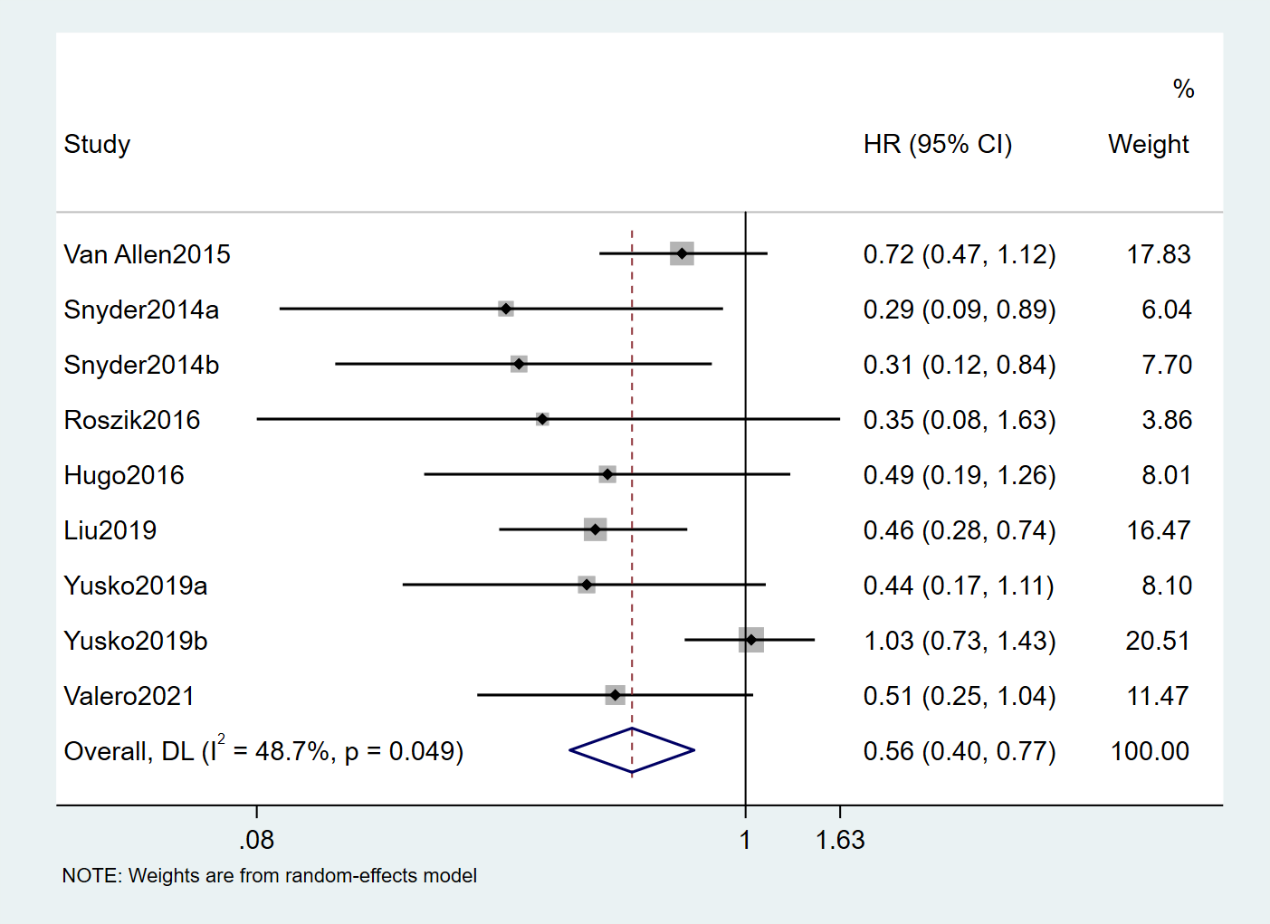


Supplementary Figure 1. Forest plot of association between TMB and OS after modification.


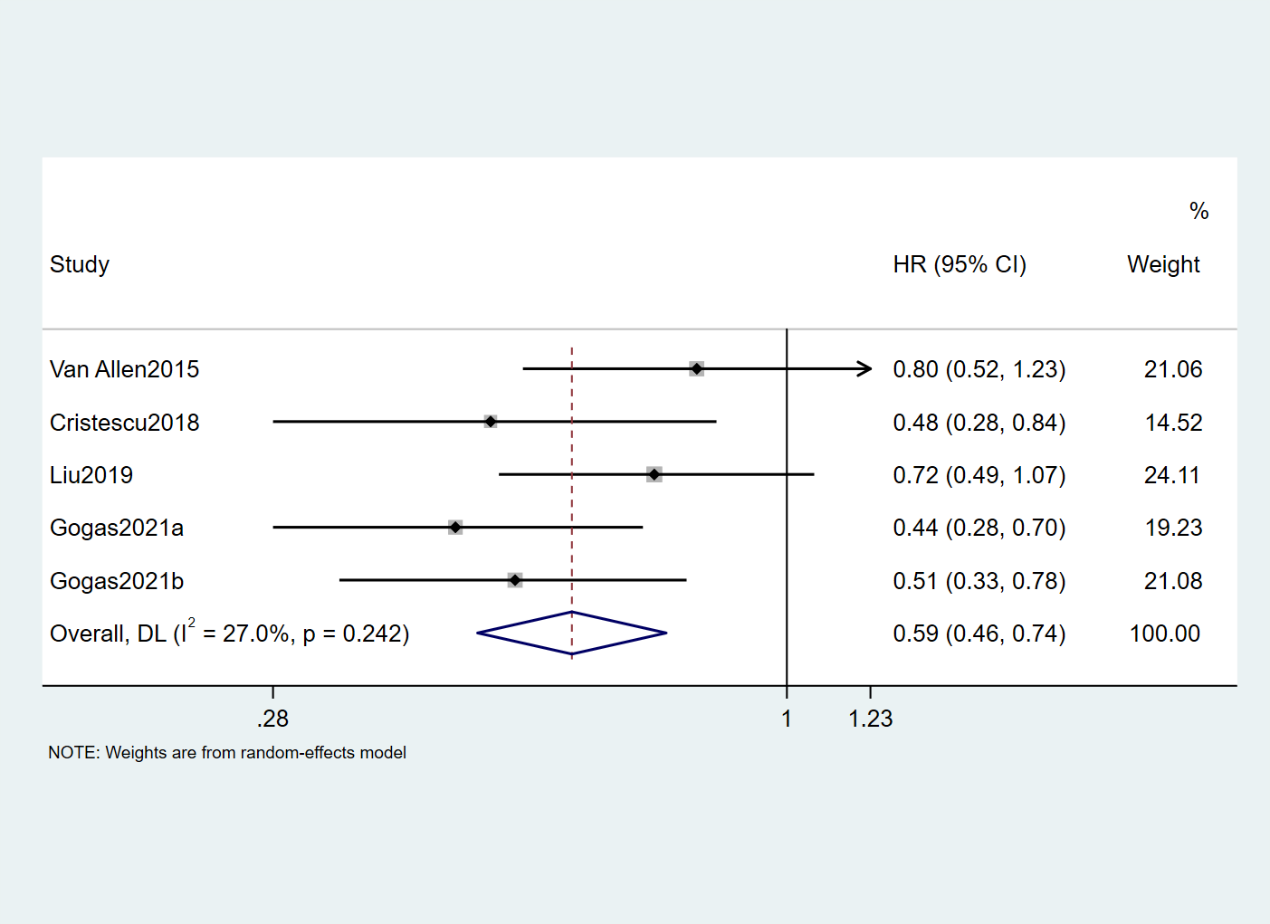


Supplementary Figure 2. Forest plot of association between TMB and PFS after modification.


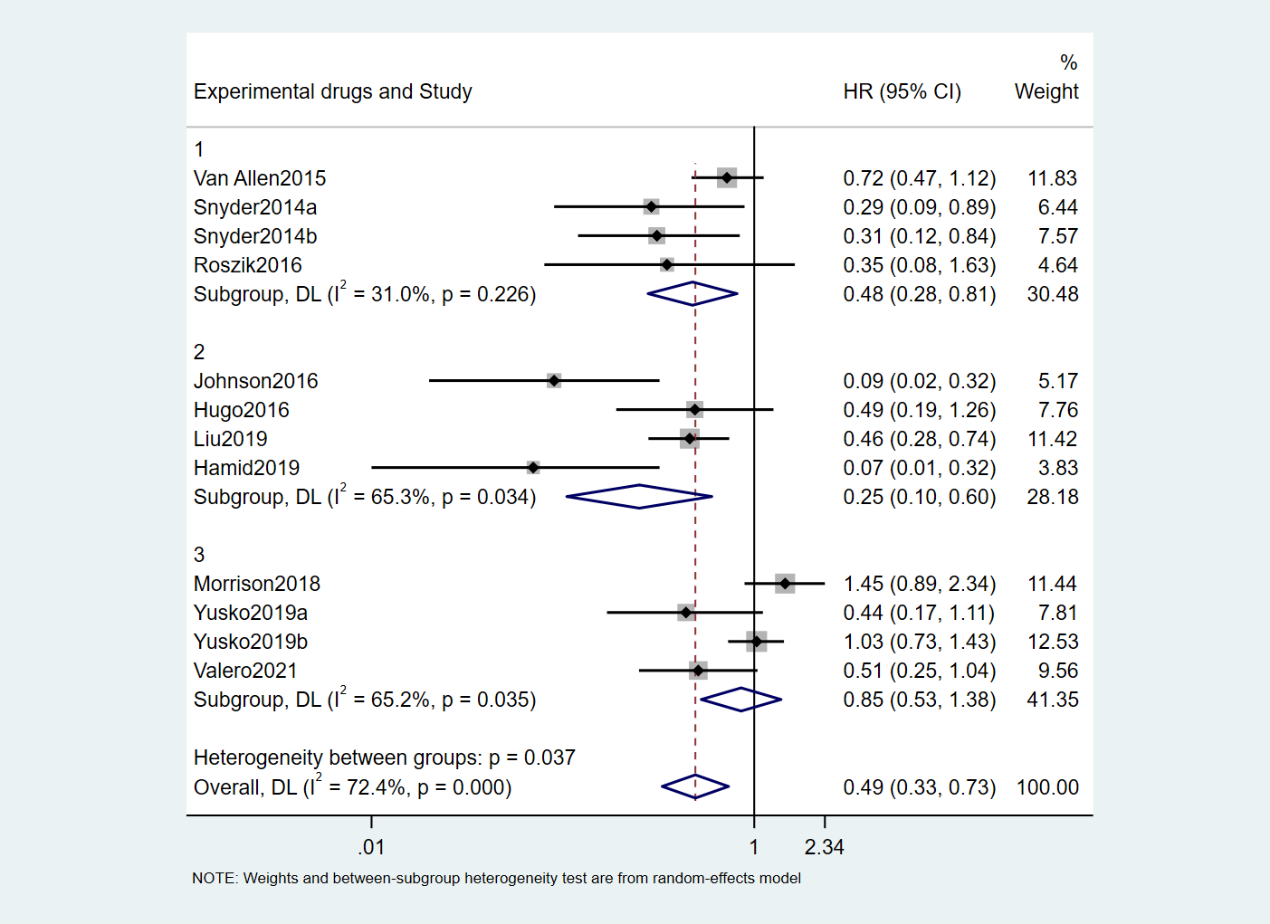


Supplementary Figure 3. Subgroup analysis for OS in melanoma patients assigned to experimental drugs. HR: Hazard Ratio.


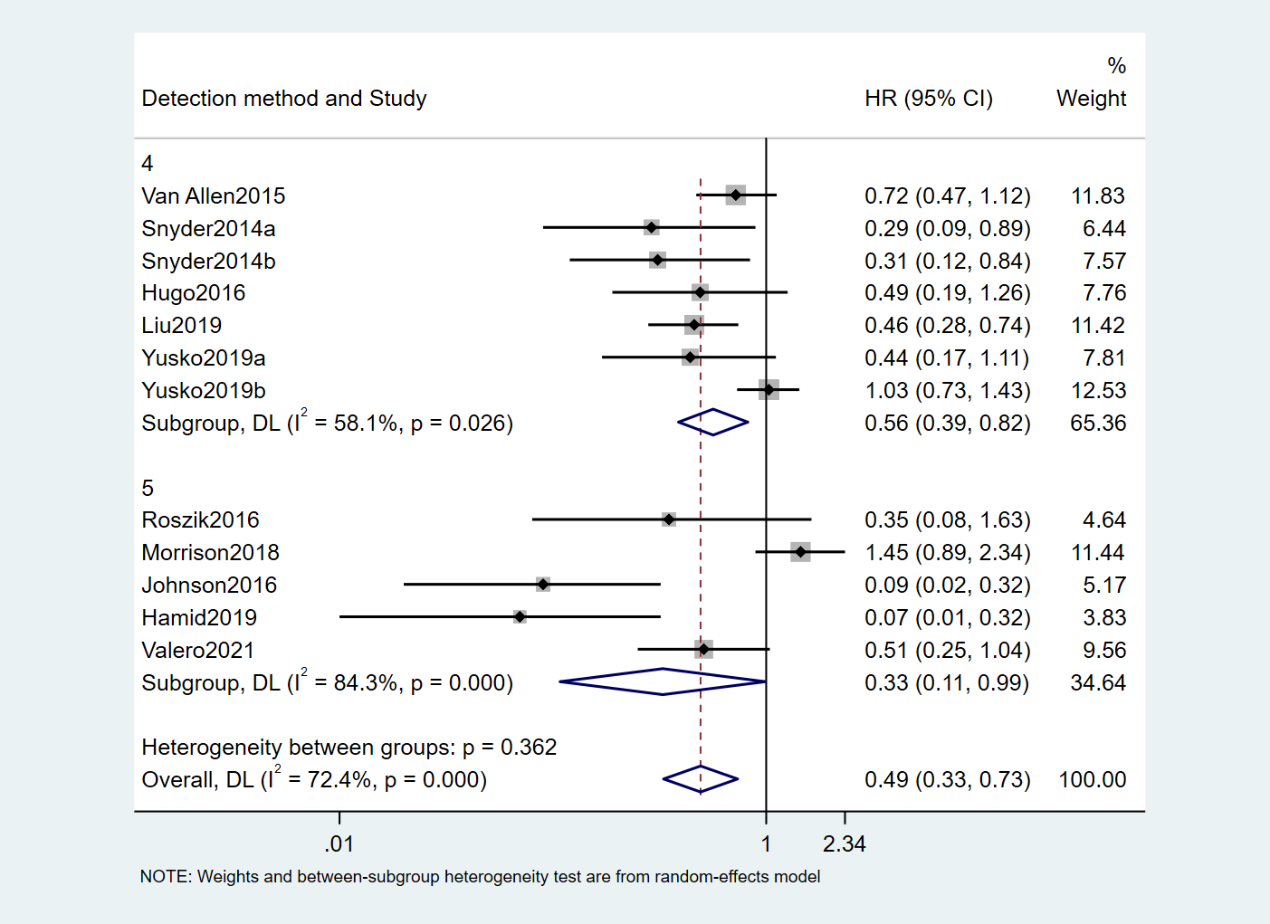


Supplementary Figure 4. Subgroup analysis for OS in melanoma patients assigned to detection method. HR: Hazard Ratio.


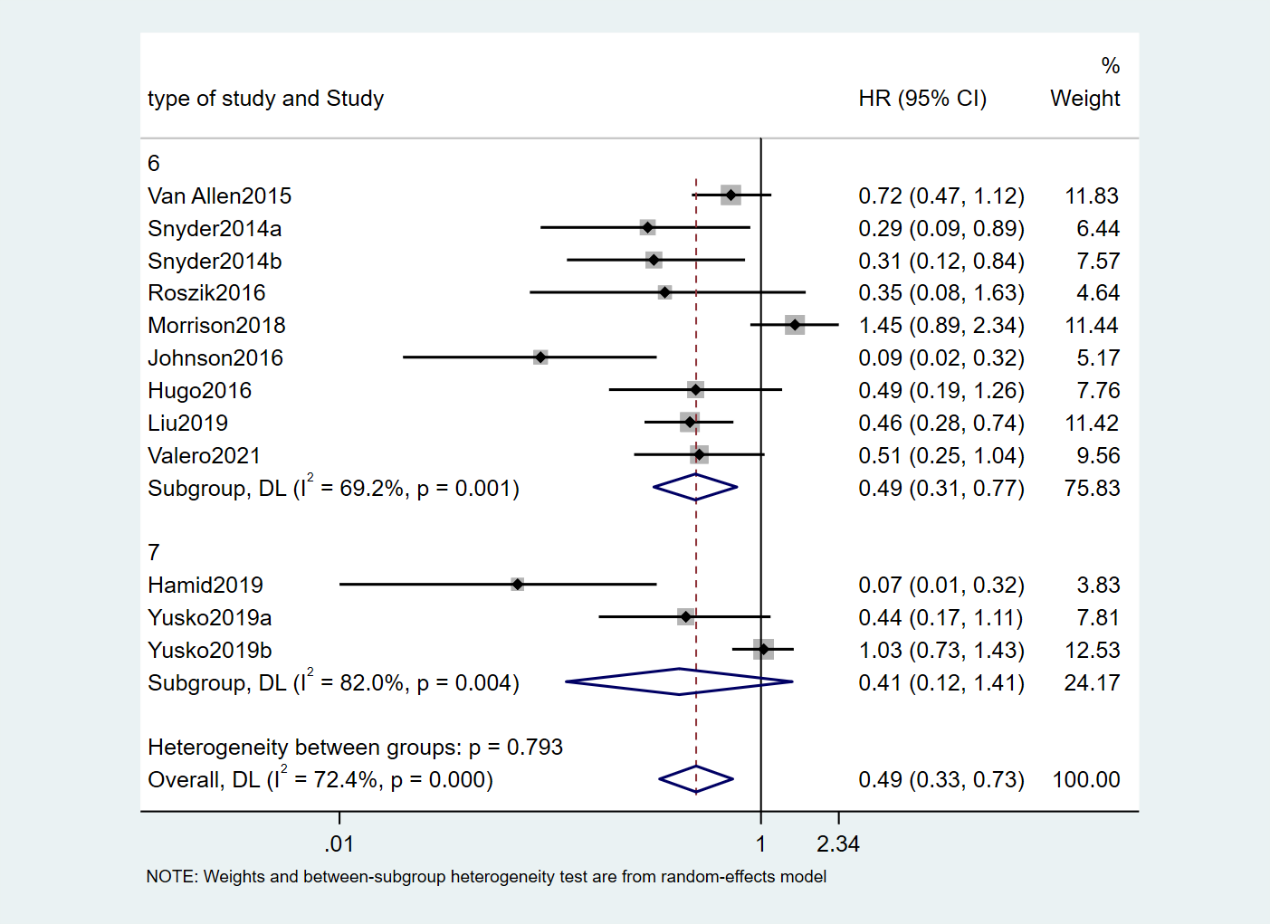


Supplementary Figure 5. Subgroup analysis for OS in melanoma patients assigned to type of study. HR: Hazard Ratio.


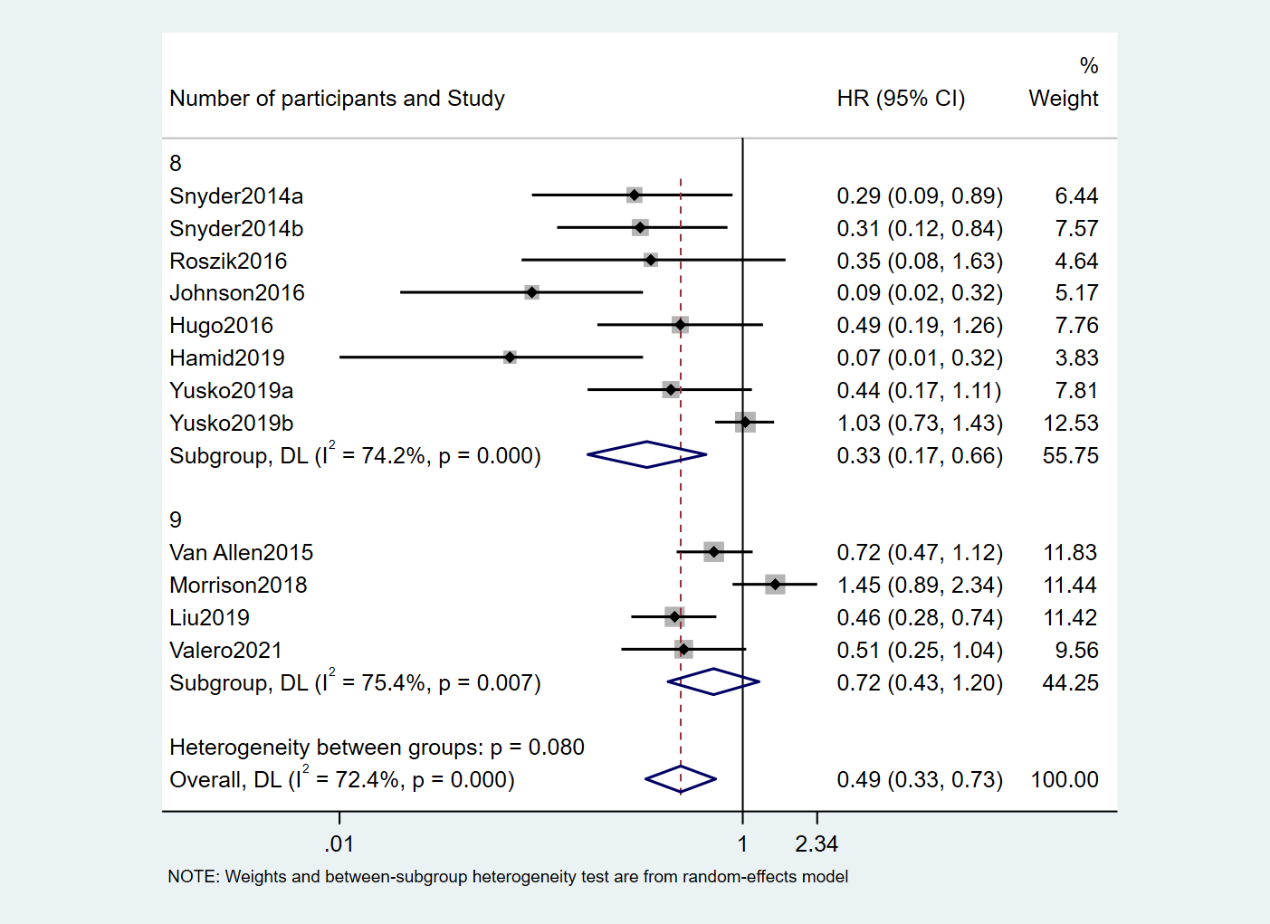


Supplementary Figure 6. Subgroup analysis for OS in melanoma patients assigned to number of participants. HR: Hazard Ratio.


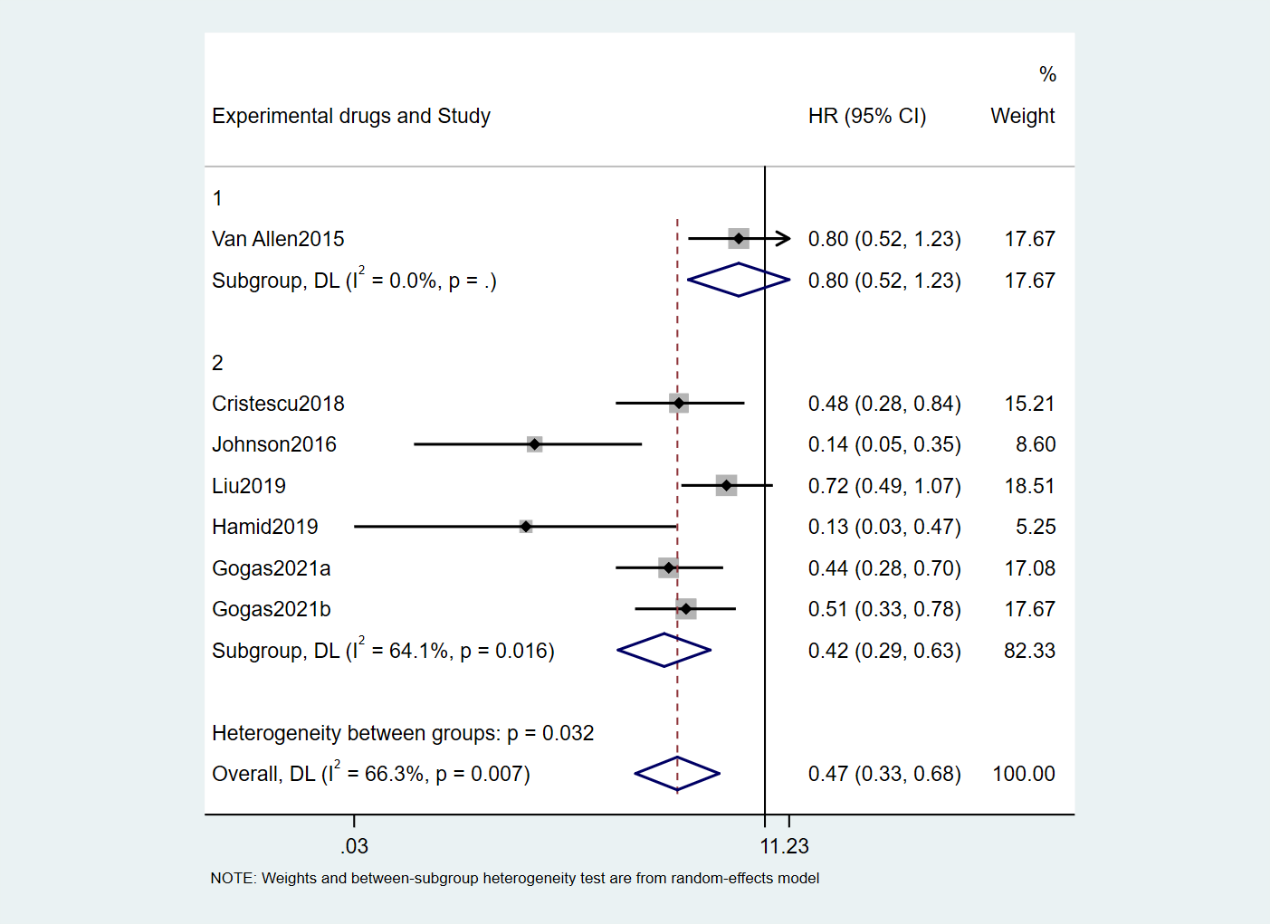


Supplementary Figure 7. Subgroup analysis for PFS in melanoma patients assigned to experimental drugs. HR: Hazard Ratio.


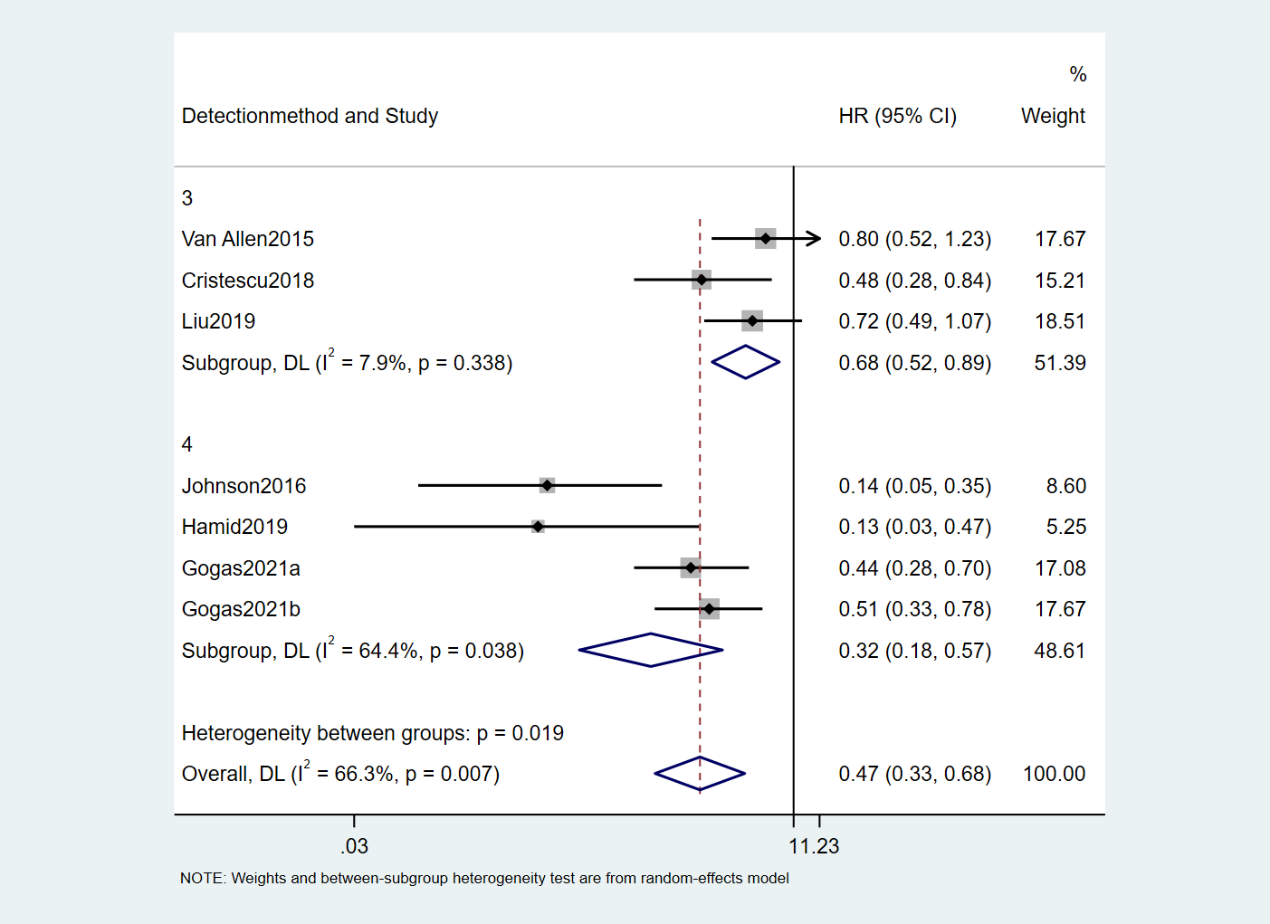


Supplementary Figure 8. Subgroup analysis for PFS in melanoma patients assigned to detection method. HR: Hazard Ratio.


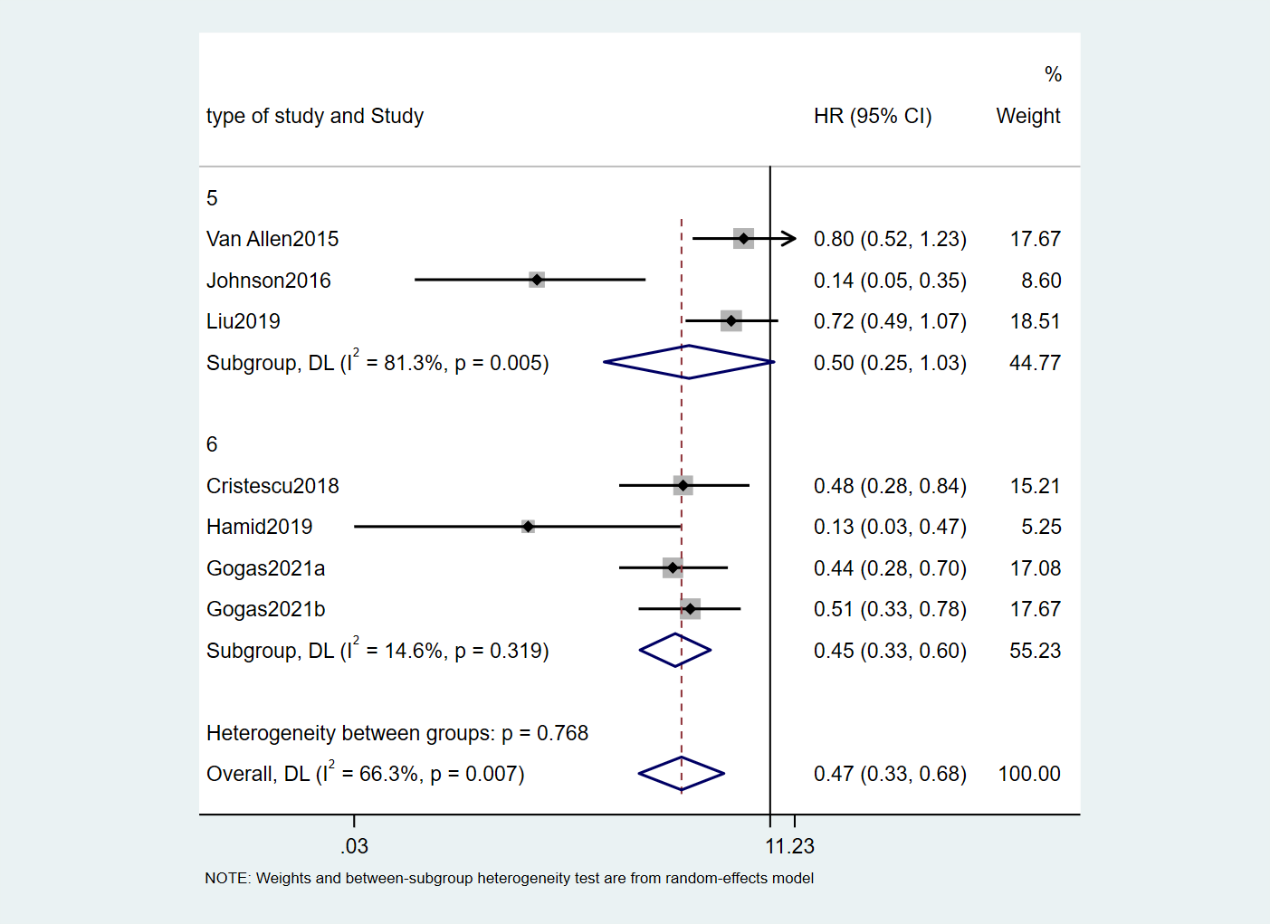


Supplementary Figure 9. Subgroup analysis for PFS in melanoma patients assigned to type of study. HR: Hazard Ratio.


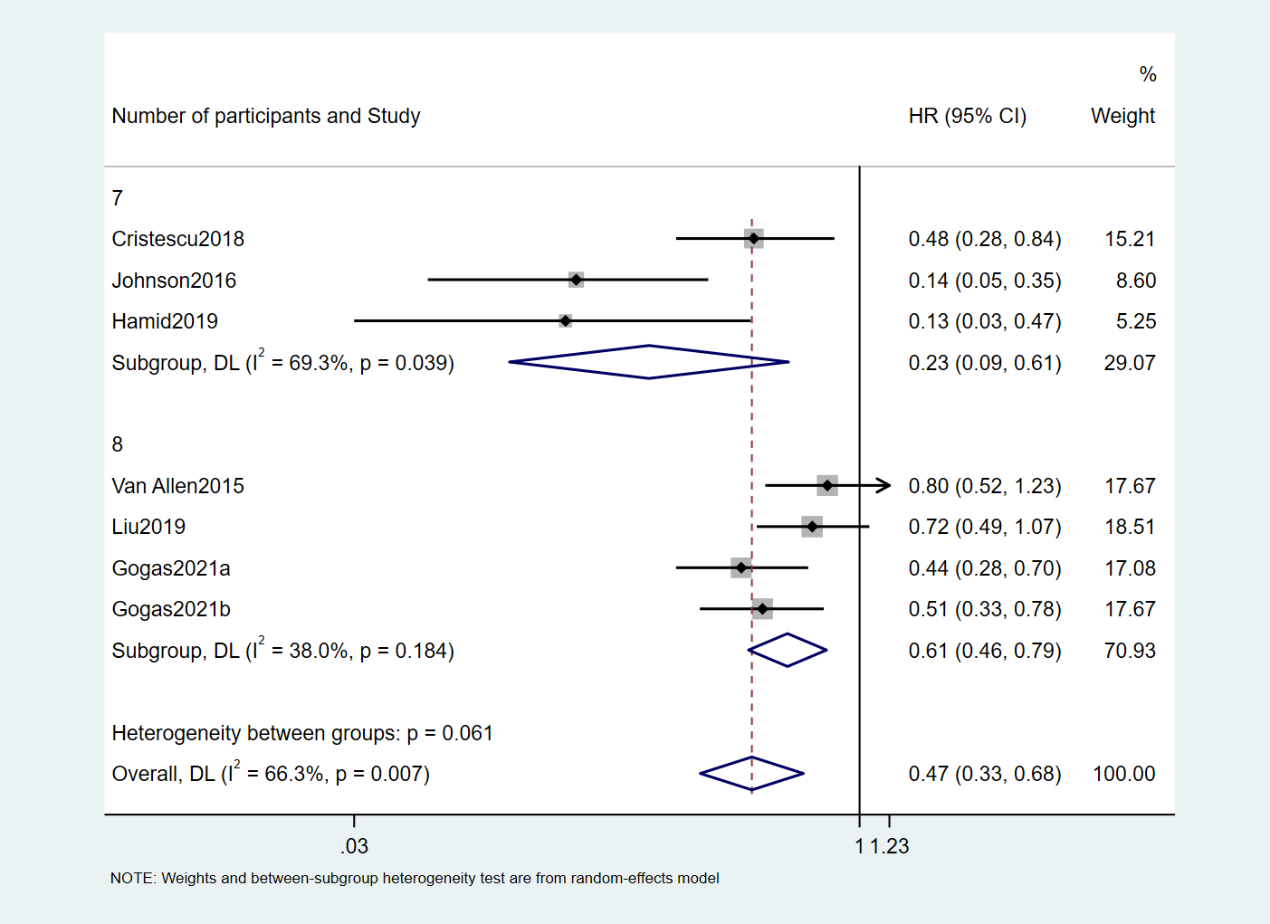


Supplementary Figure 10. Subgroup analysis for PFS in melanoma patients assigned to number of participants. HR: Hazard Ratio.
